# Using cell sheets to regenerate mouse submandibular glands

**DOI:** 10.1038/s41536-019-0078-3

**Published:** 2019-07-04

**Authors:** Kihoon Nam, Kyungsook Kim, Spencer M. Dean, Callie T. Brown, Ryan S. Davis, Teruo Okano, Olga J. Baker

**Affiliations:** 1 School of Dentistry, The University of Utah, Salt Lake City, UT USA; 2 Cell Sheet Tissue Engineering Center (CSTEC), Department of Pharmaceutics and Pharmaceutical Chemistry, The University of Utah, Salt Lake City, UT USA; 3 Institute of Advanced Biomedical Engineering and Science, Tokyo Women’s Medical University, Tokyo, Japan

**Keywords:** Regenerative medicine, Preclinical research

## Abstract

Temperature-responsive polymer grafted tissue culture dishes release cells as confluent living sheets in response to small changes in temperature, with recovered cell sheets retaining cell–cell communications, functional extracellular matrices and tissue-like behaviors. These features promote tissue regeneration and improve transplantation efficacy in various tissues including cartilage, heart, kidney, liver, endometrium, cornea, middle ear, periodontium, and esophageal living sheet transplants. However, the functional effects of cell sheets for salivary gland regeneration to treat hyposalivation have not yet been studied. Thus, the present study aims to both establish the viability of thermoresponsive cell sheets for use in salivary glands and then explore the delivery option (i.e., single vs. multiple layers) that would result in the most complete tissue growth in terms of cell differentiation and recovered tissue integrity. Results indicate that single cell sheets form polarized structures that maintain cell–cell junctions and secretory granules in vitro while layering of two-single cell sheets forms a glandular-like pattern in vitro. Moreover, double layer cell sheets enhance tissue formation, cell differentiation and saliva secretion in vivo. In contrast, single cell sheets demonstrated only modest gains relative to the robust growth seen with the double layer variety. Together, these data verify the utility of thermoresponsive cell sheets for use in salivary glands and indicates the double layer form to provide the best option in terms of cell differentiation and recovered tissue integrity, thereby offering a potential new therapeutic strategy for treating hyposalivation.

## Introduction

Xerostomia is the sensation of having a dry mouth and is commonly associated with a reduction of saliva flow (i.e., hyposalivation).^1,2^ Several conditions have been linked to hyposalivation including the following: (a) Sjögren’s syndrome, the second most common rheumatic disease after rheumatoid arthritis affecting more than 4 million people in the US,^3,4^ (b) γ-irradiation therapy that affects ~60,000 head and neck cancer patients in the US,^5^ (c) genetic diseases including lacrimo-auriculodentodigital syndrome, autosomal dominant salivary gland hypoplasia, salivary glands agenesis and chronic recurrent sialadenitis that are less frequent but cause a significant burden in many patients,^6,7^ and (d) side effects of more than 50 commonly used medications.^8,9^ Causes of hyposalivation are thus seen to be multiple but with presenting symptoms typically including unresolved inflammation and/or impaired tissue homeostasis and regeneration.^10^ Moreover, hyposalivation is a known risk factor for a variety of other serious oral health conditions including tooth decay, oral mucositis, and fungal infections.^11,12^

Current treatments for hyposalivation include the use of artificial saliva and secretory agonists such as pilocarpine and cevimeline; however, they provide only temporary relief and result in significant side effects, respectively.^13,14^ In the search for novel alternatives, some promise has been shown with the use of gene therapy for aquaporin 1 (AQP1) directed to improve water secretion in ductal cells. Although this therapy has indeed proven to be somewhat successful in addressing hyposalivation, it still does not offer a replacement for salivary proteins.^15,16^ Other studies pursuing alternative treatments include the use a variety of scaffolds using natural (e.g., fibrin hydrogel,^17–19^ collagen,^20^ hyaluronic acid,^21^ silk,^22^ and alginate^23^) as well as synthetic polymers (e.g., poly-glycolic acid, poly-lactic acid, and polyethylene glycol^24–26^), all of which have been shown to promote salivary gland regeneration, both in vitro and in vivo. Nonetheless, the longstanding problem in this area is how to achieve growth (i.e., proliferation of differentiated tissue) without that growth becoming uncontrolled (i.e., development of tumorigenic cells), and studies are ongoing in an attempt to achieve such a balance with each of the scaffolds listed above. Additional efforts have involved the use stem cells,^27–29^ which appear to promote glandular regeneration; however, this therapy alone is insufficient to adequately treat hyposalivation for two reasons. First, it is difficult to transplant stem cell suspensions into a targeted area, as they disperse throughout the tissue once injected, which in turn can lead to dilution of cell suspension into the blood stream and a related decrease in regenerative effects.^30,31^ Additionally, the use of proteolytic enzymes during stem cell isolation damages extracellular matrix proteins and may result in loss of differentiated phenotype.^32^ Despite the shortcomings of stem cells noted above, this technology nonetheless remains very attractive due to the possibility of providing for a steady stream of host tissue.

Emerging cell sheet technology, in which cells are transplanted onto a thermoresponsive plate that rapidly attach to desired tissue and subsequently merge with that target tissue, offers just such sourcing advantages without the risks associated with stem cells, as noted above.^33,34^ Moreover, this technology has been employed extensively in a wide variety of organs (e.g., cartilage,^35^ heart,^36^ kidney,^37^ liver,^38^ endometrium,^39^ cornea,^40^ middle ear,^41^ periodontium,^42^ and esophageal living sheet transplants^43^). However, cell sheets have not yet been demonstrated to be effective in salivary glands (i.e., they have not been transplanted and shown to induce salivary gland regeneration) and, beyond the issue of viability of the procedure itself, technical questions related to the particular constitution of the cell sheets to be used (e.g., single vs. multiple layers) must also be resolved. Specifically, early studies demonstrated that single cell sheet implantation can be used for repairing periodontal ligament,^44^ skin^45^ as well as corneal^40^ and bladder tissues,^46^ while later studies suggest that multiple layer cell sheets may be even more effective for repairing these same tissues^47–49^ in addition to myocardium,^50^ smooth muscle,^51^ liver,^38^ and other 3D tissues.^52^ Thus, the present study aims to both establish and then later test the viability of thermoresponsive cell sheets for use in salivary glands and then explore the delivery option (i.e., single vs. multiple layer cell sheets) that would result in the most complete tissue growth in terms of cell differentiation and recovered tissue integrity.

## Results

### Submandibular gland (SMG) cells form polarized cell sheets in vitro

**Fig. 1:**
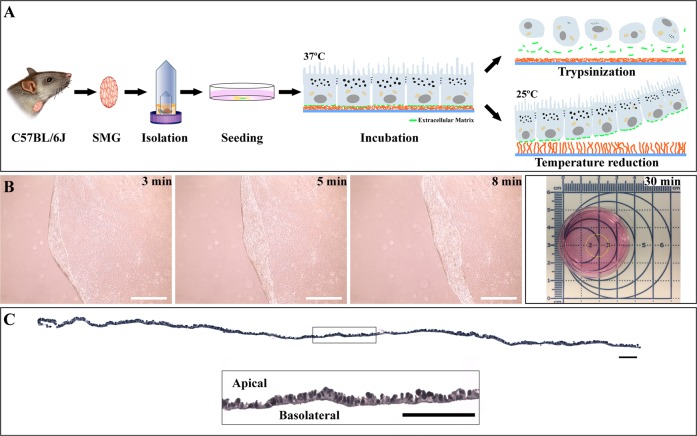
SMG cells form single sheets. **a** SMG tissue was dissociated using a GentleMACS and plated on a thermoresponsive culture dish at 37 °C for eight days, as described in Methods. Dish temperature was then reduced to 25 °C, which in turn caused the cells to detach from the surface and subsequently maintained intact the extracellular matrix proteins as compared to a traditional cell isolation method using trypsin, in which these proteins quickly disperse. **b** Sequence of cells detaching from the thermoresponsive plate depicted at 3, 5, and 8 min, with complete detachment occurring after 30 min. White bars represent 200 µm. **c** Single layer cell sheets were embedded in paraffin, sectioned, stained with H&E and imaged using a Leica DMI6000B inverted microscope at 10×. Black bars represent 100 µm. The diagram was drawn by author Kihoon Nam

To investigate whether freshly isolated mouse SMG cells were able to form a cell sheet, cells were cultured on a polystyrene dish covalently covered with a temperature-responsive polymer poly(*N*-isopropylacrylamide) (PIPAAm) (i.e., thermoresponsive cell culture dish) for eight days as described in Methods and depicted in Fig. 1a. Our results showed that cells form a single layer capable of detaching from the culture dish when the temperature is decreased from 37 °C to 25 °C (Fig. 1b), while a single layer cell sheet (Fig. 1c) likewise displayed a closely packed columnar pattern with a flat basolateral side and a protrusive apical side (Fig. 1c). Together, these results demonstrate that SMG are capable of forming polarized cell sheets in vitro when cultured on thermoresponsive plates.

### Single layer cell sheets maintain tight junctions (TJ) and secretory granules (SG) in vitro

**Fig. 2:**
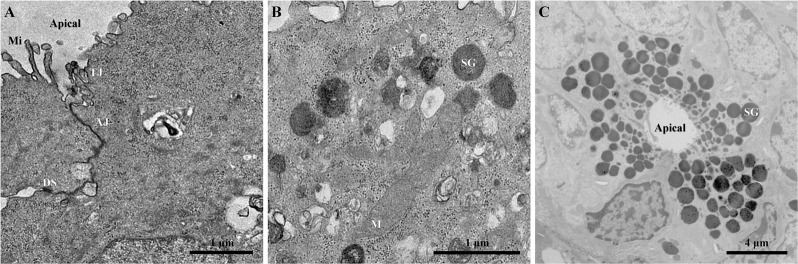
SMG-derived single layer cell sheets maintain tight junctions and secretory granules. Shown are transmission electron micrographs (TEM) of SMG cells grown on thermoresponsive plates for 8 days. Cells were processed for morphological analysis, as described in the “Methods” section and cell junctions (**a**) and secretory granules (**b**) detected and compared to native SMG (**c**). Data are representative of results from three experiments. Microvilli (Mi), tight junction (TJ), adherence junction (AJ), desmosomes (DS), secretory granules (SG)

Since SMG cells formed single layer sheets, we determined whether they displayed a polarized secretory phenotype as well. As shown in Fig. 2a, single cell sheets were able to form structures consistent with intercellular junctions including tight junctions (TJ), adherence junctions (AJ) as well as desmosomes (DS). Additionally, we detected microvilli-like structures on the apical side of the cell sheet (Fig. 2a) and SG located towards the apical membrane (Fig. 2b) similar to mouse native SMG specimen (Fig. 2c). Thus, a SMG-derived single layer cell sheet has features consistent with polarized secretory epithelia.

### Single layer cell sheets polarize and differentiate in vitro

**Fig. 3:**
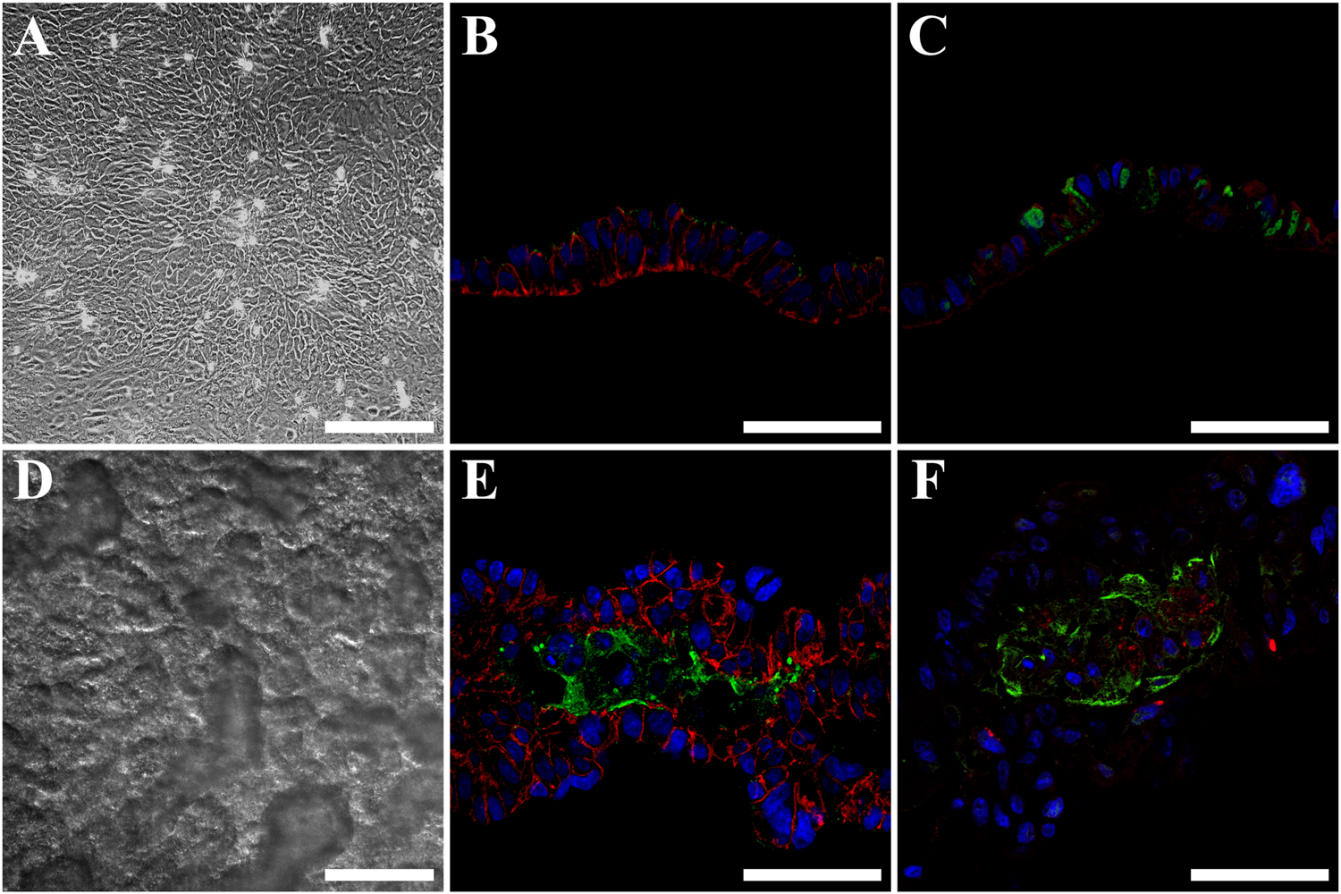
Single layer cell sheets polarize and differentiate while double layer cell sheets form a glandular-like appearance. SMG-derived single or double cell sheets were imaged using a differential interference contrast microscopy (**a**, **d**) scale bars = 200 μm as well as using a Carl Zeiss 700 confocal microscope at 40× (scale bars = 50 μm) with the following specifications rabbit anti-ZO-1 (**b** and **e**, green) and mouse anti-E-cadherin (**b** and **e**, red), rabbit anti-aquaporin 5 (**c** and **f**, green) and F-actin (**c** and **f**, red). Data are representative of results from three experiments

To confirm the presence of TJ proteins and detect markers of SMG differentiation, we analyzed cell sheet sections by confocal microscopy. Our results showed that single layer cell sheets display a columnar epithelial-like arrangement (Fig. 3a) expressing epithelial junctions including the apical TJ protein zonula occludens-1 (ZO-1, Fig. 3b, green) as well as the basolateral protein E-cadherin (Fig. 3b, red). Moreover, single layer cell sheets expressed the salivary gland acinar marker aquaporin 5 (AQP5, Fig. 3c, green) with F-actin (Fig. 3c, red). These results suggest that SMG cells are capable of forming single layer sheets that polarize and differentiate but do not display the typical three-dimensional glandular-like arrangements of a salivary gland.

### Double layer cell sheets form a glandular-like appearance in vitro

Since single cell sheets did not form a glandular appearance, we investigated whether a combination of two-single layer cell sheets promotes formation of a glandular-like appearance tissue in vivo. As shown in Fig. 3d, placing two-single cell sheets on top of each other for one day led to the formation of a double layer cell sheet with a glandular-like appearance in which the majority of cells displayed organized round structures consistent with epithelial lumens, as indicated by the intense ZO-1 staining on the apical region (Fig. 3e). Moreover, acinar differentiation marker expression, AQP5, was significantly increased after one day of layering (Fig. 3f). Together, these results indicate that double layer cell sheets display a more organized pattern as compared to single cell sheets and therefore could be used for in vivo studies.

### Double layer cell sheets show a higher intracellular calcium signaling in response to carbachol in vitro

**Fig. 4:**
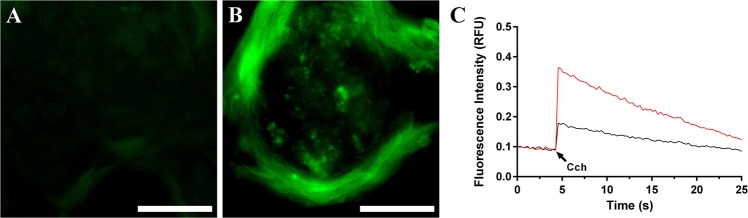
Double layer cell sheets display a more robust functionality. Both **a** single and **b** double layer cell sheets were stimulated with carbachol (Cch, 100 μM). **c** Then, changes in [Ca^2+^]_i_ were monitored in single (black line) and double (red line) layer cell sheets as described in the “Methods” section. Data are representative of results from 3 experiments. White bars representing 50 μm

To determine whether cell sheets elicit intracellular calcium signaling pathways in response to secretory agonists, we investigated whether carbachol was able to induce increases in [Ca^2+^]_i_ responses cell sheets in vitro. As shown in Fig. 4, both single and double layer cell sheets displayed a significant increase in [Ca^2+^]_i_ signaling in response to carbachol. However, double layer cell sheets (Fig. 4b) showed a significantly higher intracellular calcium increase as compared to single layer cell sheets (Fig. 4a). Together, these results indicate that while both single and double cell sheets are functional, double layer cell sheets show a stronger agonist-mediated response (Fig. 4c).

### Double layer cell sheets promote tissue organization in vivo

**Fig. 5:**
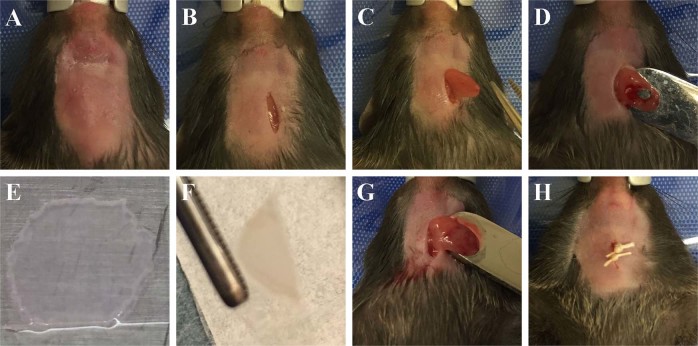
Double layer cell sheets can be directly transplanted to wounded SMGs. Skin incisions of approximately 1 cm in length were made along the anterior surface of the neck of a C57B/L6J and SMG were exposed (**a**–**c**). Then, a 3 mm diameter biopsy punch was performed and **d** surgical wounds filled with a single or double layer cell sheet measuring approximately 1 cm of diameter of a semicircle (**e**–**g**). Finally, the skin incision was sutured (**h**) and mice were placed in a recovery room

**Fig. 6:**
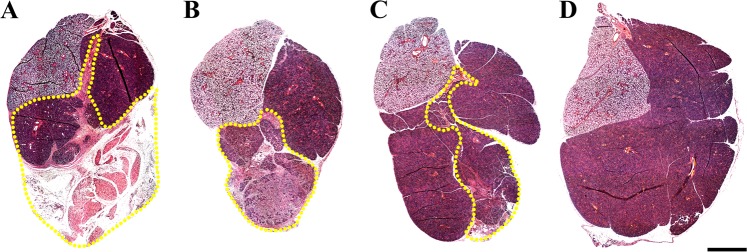
Double layer cell sheets promote tissue organization to a similar extent as sham controls. H&E staining of wounded SMG that **a** remained untreated, **b** were treated with single cell sheets, **c** were treated with double cell sheets or **d** were unwounded (sham controls) was performed and specimens were analyzed by light microscopy using a Leica DMI6000B as described in the “Methods” section; bars = 1 mm with yellow dotted areas indicating wounded areas. Data are representative of results from three experiments

Given that double layer cell sheets formed a glandular-like structures in vitro, we decided to use double layer cell sheets for regeneration studies in vivo by transplanting SMG double layer cell sheets into a wounded mouse model (Fig. 5), as described in the “Methods” section. Our results indicate that untreated SMG surgical wounds displayed fibrotic tissue (Fig. 6a) at post-surgery day 8. In contrast, SMG surgical wounds treated with a single layer cell sheet displayed almost complete wound closure, exhibited signs of fibrosis and partial wound healing (Fig. 6b). Interestingly, SMG surgical wounds treated with a double layer cell sheet (Fig. 6c) displayed an approximately 90% wound closure with a similar morphology to sham controls (Fig. 6d). Together, the histological results demonstrate that a double layer cell sheet promotes regeneration in wounded mouse SMG after eight days.

### Double layer cell sheets promote tissue differentiation in vivo

**Fig. 7:**
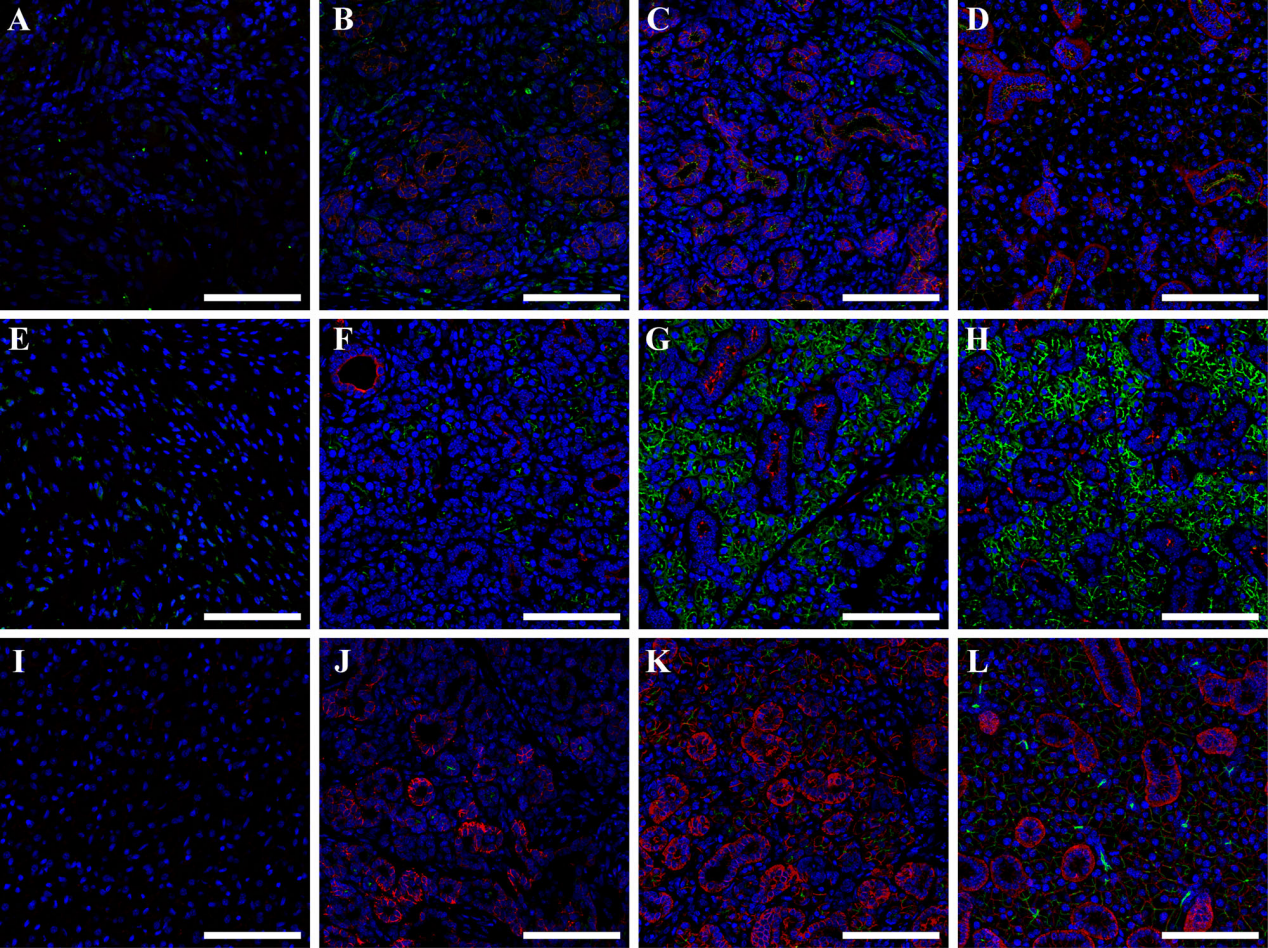
Double layer cell sheets promote tissue polarization and differentiation to a similar extent as sham controls. Confocal analysis of wounded SMG that remained untreated (**a**, **e**, **i**), were treated with single cell sheets (**b**, **f**, **j**), were treated with double cell sheets (**c**, **g**, **k**) or were unwounded (sham controls, **d**, **h**, **l**) was performed as follows: rabbit anti-ZO-1 (**a**–**d**; green) and mouse anti-E-cadherin (**a**–**d**; red), rabbit anti-aquaporin 5 (**e**–**h**; green) and mouse anti-cytokeratin 7 (**e**–**h**; red), rabbit anti-TMEM16A (**i**–**l**; green) and mouse anti-Na^+^/K^+^ ATPase (**i**–**l**; red). Data are representative of results from 5 experiments. White bars represent 100 µm

Since a double layer cell sheet promotes regeneration in wounded mouse SMG, we determined whether the newly formed tissue displayed polarity and differentiation under these conditions. Our results show that untreated wounded SMG expressed both ZO-1 (Fig. 7a, green) and E-cadherin (Fig. 7a, red), with either protein expressing only a very weak staining together with a disorganized structure. In contrast, SMG treated with a single layer cell sheet expressed slightly organized apical ZO-1 (Fig. 7b, green) and basolateral E-cadherin (Fig. 7b, red). Interestingly, SMG treated with a double layer cell sheet expressed ZO-1 (Fig. 7c, green) and E-cadherin (Fig. 7c, red) with a strong apical and basolateral staining respectively, similar to sham controls (Fig. 7d, green and red) and indicating polarity. Moreover, untreated SMG barely expressed the acinar marker AQP5 (Fig. 7e, green) or the ductal marker cytokeratin 7 (Fig. 7e, red), with both proteins showing a weak staining and a disorganized pattern. In contrast, SMG treated with a single layer cell sheet partially expressed AQP5 (Fig. 7f, green) and cytokeratin-7 (Fig. 7f, red). Also, SMG treated with a double layer cell sheet expressed AQP5 (Fig. 7g, green) and cytokeratin-7 (Fig. 7g, red), where both proteins displayed strong apical and basolateral staining patterns, respectively, similar to sham controls (Fig. 7h, green and red) indicating differentiation. Likewise, untreated wounded SMG did not express the acinar marker transmembrane protein 16 (TMEM16, Fig. 7i, green) or the functional basolateral marker sodium potassium ATPase (Na^+^/K^+^-ATPase, Fig. 7i, red), with both proteins showing no staining. Conversely, SMG treated with a single layer cell sheet expressed weak apical TMEM16 (Fig. 7j green) and strong basolateral Na^+^/K^+^-ATPase (Fig. 7j, red), Finally, SMG treated with a double layer cell sheet expressed strong apical TMEM16 (Fig. 7k green) and basolateral Na^+^/K^+^-ATPase (Fig. 7k, red), similar to sham controls (Fig. 7l, green and red) indicating functionality.

### Double layer cell sheets promote saliva secretion in vivo

**Fig. 8:**
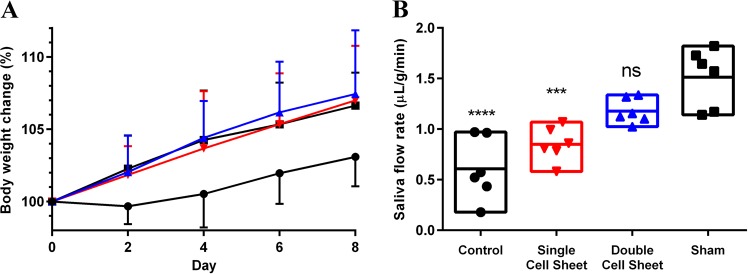
Double layer cell sheets restore body weight and saliva secretion to a similar extent as sham controls. **a** After SMG wounds were made, mice received the indicated treatments and changes in body weight (%) of untreated (●), single cell sheet () or double cell sheet () treated mice groups were compared with sham control group (■) over 8-day period. Data represent the means ± SD of *n* = 9 mice per condition where statistical significance was assessed by two-way ANOVA (*P* *<* 0.01) and Dunnett’s post hoc test for multiple comparisons to the sham group. **b** After the various treatments, mice were anesthetized and stimulated with pilocarpine and isoproterenol as described in the “Methods” section. Then, saliva was collected for 5 min. Data represent the means ± SD of *n* = 6 mice per condition and statistical significance was assessed by one-way ANOVA (ns not significant; ***P < 0.001 and ****P < 0.0001) and Dunnett’s post hoc test for multiple comparisons to the sham control groups (*)

Our previous studies indicated a decrease in saliva flow rates wounded mouse SMG.^18,19^ Since saliva is critical for eating and swallowing, we determined the capability of the mice to perform these functions by measuring their body weight at various post-surgery times. Our results show that untreated mice exhibited a significant decrease in body weight (Fig. 8a). In contrast, mice treated with a single or double layer cell sheet displayed body weights comparable to sham controls (Fig. 8a). Since a double layer cell sheet promoted regeneration in wounded mouse SMG as compared to controls, we determined whether the newly formed tissue improved salivary secretory function under these conditions. Our results show that untreated mice exhibited a significant decrease in saliva flow rates (Fig. 8b). In contrast, mice treated with a double layer cell sheet exhibited saliva flow rates comparable to sham controls (Fig. 8b).

## Discussion

Given the success of cell sheets for tissue regeneration in a variety of organs (e.g., cartilage, heart, kidney, liver, endometrium, cornea, middle ear, periodontium, and esophageal living sheet transplants),^35–52^ they can be applied in salivary glands to promote cell differentiation and tissue integrity. Previous studies have shown that cell sheets can be used to enhance regeneration in a variety of organs as well as in multiple animal models.^44,53–55^ Additionally, clinical trials are ongoing to use cell sheets for treatment of esophageal stricture, ischemic heart disease, cholesteatoma, cardiomyopathy, ocular disease and air leaks.^53,56–62^ In light of the above findings as well as the current lack of effective treatments for hyposalivation, it would appear that cell sheet technology could thus present a viable alternative treatment to promote salivary gland regeneration and restore secretory function.

Some of the advantages of SMG cell sheets for in vitro studies is that cells grown under these conditions can be cultured as monolayers while maintaining all intrinsic ECM proteins, TJs and SG (Fig. 2). Additionally, double layer cell sheets are able to form acinar and ductal-like organoids with a three-dimensional shape containing lumens and consistent with salivary gland epithelium (Fig. 3) that respond to secretory agonists (Fig. 4). These features offer an improvement over freshly isolated SMG cells plated on plastic where they lose SG, form disorganized TJs and de-differentiate over time.^63^ For use of in vitro models, one could measure salivary gland cells’ ability to form cell sheets under conditions such as pro-inflammatory cytokine exposure or irradiation as well as perform phosphorylation and protein expression studies (given that these organoids are similar to native tissue).^64^ Likewise, each of these functions could in turn be measured in cell sheets derived from human SMG, given that such tissue is relatively easy to obtain and culture.^65^ Together, our studies demonstrate that cell sheets could be used as a good in vitro model for studying salivary gland cell signaling and function. Finally, apparent limitations of cell sheets for in vitro studies were noted, including their inability to be used for measurement of saliva secretion, the possibility of losing proteins during homogenization and their limited lifespan in culture due to lack of irrigation and innervation; nonetheless, these complicating factors should not significantly reduce the value of this model.

Regarding the advantages of cell sheets for in vivo studies, autologous cells could be used for transplantation, as cells sheets completely detach from temperature-responsive plate without carrying any chemicals or contaminants.^66–71^ Moreover, because cell sheets are able to maintain ECM proteins, they can rapidly attach to target organ surfaces without needing sutures, thereby facilitating regenerative treatments.^66–71^ In this regard, our results showed that a double layer cell sheet optimizes tissue formation (Fig. 6), cell differentiation (Fig. 7) and saliva secretion (Fig. 8). In contrast, single cell sheet-treated did not show these improvements. These results suggest that pre-formed cellular interactions within double layer cell sheets may help the tissue to differentiate by delivering paracrine factors while promoting functionality. Since single cell sheet-treated mice showed an improvement in weight (Fig. 8a) it is also possible that this treatment is also effective in delivering paracrine factors. A noteworthy limitation of cell sheet use in vivo is the inability to completely characterize the cell types forming these sheets, with only acinar and ductal markers employed in the current study and prior studies in other organ systems typically using well characterized cell systems or choosing to focus on a single cell type. To rectify this deficiency, it is recommended that future studies concentrate on better characterizing the various cell types present within the cell sheets before implantation and during regeneration using single RNA sequencing and cell lineage tracing studies respectively.^72,73^ Furthermore, it would be beneficial to extend the regeneration studies beyond the eight days observed here to determine whether regenerative effects identified herein will be maintained and, likewise, whether this technology could be successfully applied to other in vivo models of hyposalivation (e.g., mouse models for head and neck irradiation or SS-like mice). Finally, future studies are recommended to corroborate current findings and to identify relevant mechanisms for this promising approach to salivary gland regeneration.

## Methods

### Animals

Female C57BL/6J mice 6-weeks-old, weighing ~17–20 g, were purchased from the Jackson Laboratory (Bar Harbor, ME). All animal usage, anesthesia, and surgeries were conducted with the approval of the University of Utah Institutional Animal Care and Use Committee, in accordance with their strict guidelines.

### Fresh SMG cell isolation

Mice were euthanized using carbon dioxide (CO_2_) followed by abdominal exsanguination. SMG were then removed, cut into small pieces and placed in a 35 ml GentleMACS^™^ C Tube containing 6.5% tumor dissociation enzyme mixture (Miltenyi Biotec Inc. Auburn, CA) in DMEM/F12 (Invitrogen, Carlsbad, CA). Subsequently, the tissue was dissociated using a GentleMACS (Miltenyi Biotec Inc.) and incubated in a shaking water bath at 37 °C for 30 min. After three such steps and two intervening incubations, SMG cells were centrifuged at 150 × *g* for 5 min at 4 °C and the dispersion medium was removed. The cells were then resuspended in 5 ml DMEM/F12 complete medium containing the following: 2.5% FBS, 2 nM triiodothyronine, 0.1 μM retinoic acid, 0.4 μg/ml hydrocortisone, 80 ng/ml EGF, 5 ng/ml sodium selenite, 5 mM glutamine, 5 μg/ml insulin and 5 μg/ml transferrin. Cells were then passed through 70 and 40 µm strainers (Thermo Fisher Scientific, Waltham, MA) and seeded at 1.0 × 10^6^ cells/plate on FBS coated 35-mm UpCell^™^ temperature-responsive dishes (Thermo Fisher Scientific), cultured at 37 °C in a humidified atmosphere of 95% air-5% CO_2_ and used at confluence (a time when monolayer completely covers the plate), with the cell culture medium replaced every other day.

### Cell sheet preparation

After 8 days of incubation, temperature was reduced below 25 °C for 30 min to allow the cell sheet to detach from the dish surface. After removing culture medium from single layer cell sheet, a wet transfer membrane was placed over the SMG monolayer and 20 µl of fresh medium was added to prevent the cells from drying out. After 30 min of incubation at room temperature, the attached cell layer was transferred to a new FBS coated culture dish and incubated at 37 °C for 30 min. Then, one milliliter of fresh medium was added on top of the membrane and gently removed from the cell layer. After aspirating the culture medium, the apical side of the first cell sheet layer was covered with the basolateral side of the second cell sheet and incubated at 37 °C for 30 min. Finally, the wet transfer membrane was gently withdrawn from the cell layers and a double layer cell sheet was cultured for one additional day for further experiments.

### Intracellular free calcium levels

The intracellular free calcium concentration in both single and double layer cell sheets was measured using a Leica DMI6000B imaging system. Briefly, cell sheets were transferred into a Lab Tek Chamber Slide (Thermo Fisher Scientific). After 1 day of incubation, they were treated with Fura-2-acetoxymethylester (Fura-2 AM, 4 μM) for 30 min at 37 °C in DMEM/F12 and washed two times with DMEM/F12 and two times with assay buffer (DMEM/F12 containing 10 mM glucose, 1 mM CaCl_2_, 0.1% BSA). Then, cells were stimulated with carbachol (Cch, 100 μM). Subsequently, images were recorded and analyzed using a Leica Suite X software.

### Animal model

The wounded SMG model was created following a method reported previously.^18,19^ Briefly, C57BL/6J mice were anesthetized with 3% isoflurane with an oxygen flow rate set at 2.0 L/min, SMG exposed and surgical wounds created using a 3 mm diameter biopsy punch in both glands and treated with a single or double layer cell sheet (experimental group), left untreated (untreated wounded controls) or were left unwounded (sham surgery controls). Then, the skin incision was sutured and post-surgical studies at day 8 were performed. For these purposes, SMG were dissected and processed for histological analysis and saliva secretion studies as described below.

### Hematoxylin and eosin staining

Cell sheets were fixed in 4% PFA for 10 min, dehydrated in 70% ethanol solution, embedded in paraffin wax, and cut into 3 μm sections. Then, they were deparaffinized with xylene and rehydrated with serial ethanol solutions and distilled water. Finally, hematoxylin and eosin staining was performed, and specimens were examined using a Leica DMI6000B inverted microscope (Leica Microsystems, Wetzlar, Germany).

### Saliva flow rate measurements

To collect stimulated saliva secretion, mice were anesthetized with ketamine (100 mg/kg) and xylazine (5 mg/kg), and injected with pilocarpine (50 mg/kg) and isoproterenol (0.5 mg/kg) via intraperitoneal injection.^74,75^ Then, stimulated saliva was collected using a micropipette for 5 min.^19^ Finally, statistical significance was assessed by one-way ANOVA (*P* *<* 0.01) and Dunnett’s post hoc test for multiple comparisons to the untreated group and sham control group.

### Weight change

Mice were weighed at the start of each experiment and data was collected for 8 days. Then, statistical significance was assessed by two-way ANOVA (*P* < 0.01) and Dunnett’s post hoc test for multiple comparisons to the untreated group.

### Transmission electron microscopy

Cells sheets or tissues were fixed overnight at 4 °C in a solution containing 2.5% glutaraldehyde, 1% paraformaldehyde, 100 mM cacodylate buffer at pH 7.4, 6 mM CaCl_2_, and 4.8% sucrose. The next day, cells were washed three times for 5 min each with cacodylate buffer, post-fixed with 2% osmium tetroxide at room temperature for 45 min, washed twice for 5 min with cacodylate buffer, then washed once with distilled water for 5 min. Specimens were then stained with saturated uranyl acetate for 45 min at room temperature, washed three times for 5 min each with distilled water, then dehydrated with consecutive ethanol washes (30%, 50%, 70%, twice at 95%, and three times with 100%) for 15 min each. This was followed by dehydration with absolute acetone three times for 10 min each. Specimens were infiltrated with consecutive EPON epoxy resin incubations (30% for 5 h, 70% overnight, three times with 100% for 8 h). 70 nm thick sections were made using a Leica Ultra Cut 6 ultratome, and imaged using a JEOL JEM-2800 operated at an accelerating voltage of 200 kV.

### Confocal analysis

A detailed procedure of deparaffinization and antigen retrieval methods of 3 μm thick paraffin embedded samples can be obtained from a previous study.^18^ Specimens were then blocked in 5% goat serum in PBS for 1 h at room temperature, and incubated at 4 °C with the primary antibodies in 5% goat serum overnight as follows: rabbit anti-ZO-1 (Invitrogen, 1:50 dilution), mouse anti-E-cadherin (BD Biosciences, San Jose, CA, 1:100 dilution), rabbit anti-aquaporin 5 (Abcam, Cambridge, MA 1:200 dilution), mouse anti-cytokeratin 7 (Abcam, 1:250 dilution), rabbit anti-TMEM16A (Abcam, 1:50 dilution) and mouse anti-Na^+^/K^+^-ATPase α antibody (Santa Cruz Biotechnology, Santa Cruz, CA1:100 dilution). Then, sections were incubated for 2 h with anti-rabbit Alexa Fluor 488 and anti-mouse Alexa Fluor 568 secondary antibody solution at 1:200 dilutions in 5% goat serum at room temperature. Subsequently, specimens were counter-stained with TO-PRO-3 Iodide nuclear stain (Invitrogen) at room temperature for 15 min at 1:1000 dilutions. Finally, specimens were analyzed using a confocal Zeiss LSM 700 microscope (Carl Zeiss, Oberkochen, Germany) at ×20 magnifications for in vivo studies and ×40 for in vitro studies. A total depth of 3 μm was acquired for each sample, and a total projection was visualized in the xy planes.

### Reporting summary

Further information on research design is available in the Nature Research Reporting Summary linked to this article.

## Supplementary information

Reporting Summary Checklist

## Data Availability

Data supporting the findings of this study are available from the corresponding author(s) upon request.

## Supplementary information

**Supplementary information** accompanies the paper on the *npj Regenerative Medicine* website (10.1038/s41536-019-0078-3).
